# Organic fertilization influences nematode diversity and maturity index in coffee tree plantations using an agroforestry system

**DOI:** 10.21307/jofnem-2021-054

**Published:** 2021-05-21

**Authors:** JOL Vieira Júnior, RC Pereira, RL Soto, IM Cardoso, EA Mondino, RLL Berbara, E Sá Mendonça

**Affiliations:** 1 Department of Entomology and Plant Pathology, Universidade Estadual Norte Fluminense Darcy Ribeiro, Campos dos Goytacazes, Rio de Janeiro, Brazil; 2 Centro de Edafologia y Biología Aplicada del Segura, Murcia, Spain; 3 Universidade Federal de Viçosa, Viçosa, Brazil; 4 Laboratório de Nematología IPADS Balcarce (INTA-CONICET) Ruta Nac. 226 Km. 73,5-CC 276, (B7620WAP) Balcarce, Buenos Aires, Argentina; 5 Universidade Federal Rural do Rio de Janeiro, Seropédica, Rio de Janeiro, Brazil; 6 Universidade Federal do Espírito Santo, Alegre, Brazil

**Keywords:** Agroecology, Agroforestry systems, *Coffea arabica*, Ecology, Ecological indices, Organic farming, Management

## Abstract

In conventional coffee farming, soil fauna can be negatively affected by the intensive management practices adopted and the use of an agroforestry system (AFS) is an alternative to reduce these impacts. In coffee AFS, soil nutrition is provided mainly using organic fertilizers. This soil management favors the microbiota and can alter the population dynamics of some organisms. Our objective was to evaluate the effect of organic fertilizers on the nematode community in coffee AFS and to determine their impact on soil ecology. Soil samples were collected from three coffee AFS and a nearby Atlantic rainforest fragment. Nematodes were extracted from the samples and identified to the genus. The identified populations were compared using several community and diversity indices to determine the environmental conditions of the systems under evaluation. No differences in total abundance among nematode communities were found in the four areas evaluated. Regarding trophic groups, the coffee AFS treated with either cow manure or poultry litter favored the trophic group of bacterivores. Plant-parasitic nematodes were more abundant in soils of both the naturally fertilized coffee AFS and the Atlantic rainforest fragment. The maturity and structural indexes indicated that the Atlantic rainforest fragment and the naturally fertilized coffee AFS had similar ecological functions. On the other hand, soils fertilized with cow manure were less diverse, had higher dominance in the community, and showed less ecological stability. The nematode communities found in the AFS were similar to those seen in the forest fragment indicating that is possible to produce coffee sustainably without negatively affecting soil quality.

Intensive soil management practices, such as deep turnover, use of synthetic fertilizers and pesticides may lead to an imbalance in soil biodiversity and negatively affect the functionality of agroecosystems (Wall et al., 2015). Hence, some farmers have begun managing their crops in agroforestry systems (AFS) using agroecological and organic approaches to reduce the environmental impact caused by intensive agriculture. Incorporation of different tree species into coffee plantations may promote social, economic, and environmental benefits as this contributes to production diversity, increased family income, and increased organic matter in the soil (Jackson et al., 2012).

In coffee AFS, soil nutrition is provided from organic fertilizers such as cow manure, poultry litter and plant residues when necessary. The proper incorporation of these fertilizers improves soil quality and microbial activity (Jannoura et al., 2014). These fertilizers can also change the population dynamics of some organisms such as nematodes (Jannoura et al., 2014; Falkowski et al., 2019).

Soil nematodes can have different habits and are classified as either free-living (bacterivores, fungivores, predators, and omnivores) or plant-parasites (Ferris, 2010). These organisms play an important ecological role in regulating the soil microbiota, mineralization, and nutrient cycling (Bongers and Ferris, 1999). Nematodes are sensitive to changes in mulching and respond rapidly to agricultural practices such as fertilization (Ferris, 2010; Oka, 2010). Studies on the diversity of nematodes in agricultural areas have resulted in a growing interest in this field as these organisms can act as bioindicators for agroecosystems (Neher, 2001).

Depending on the fertilizer used, the availability of soil nutrients is altered and this reflects on the nematode community (Yeates et al., 2009; Zhang et al., 2019). Organic fertilization can provide nutrients for the development of bacterial-feeding nematodes and reduce the numbers of some plant-parasitic nematode species (Renco and Kovacik, 2012). Although the effects of fertilization on the abundance and diversity of soil nematodes have been widely studied, the impact of organic fertilization on complex agroecosystems, such as AFS, is unknown (Zhang et al., 2019). Thus, the objectives of this study were to (1) evaluate the effect of soil fertilization using either poultry litter, cow manure, or plant residues on nematode communities in coffee AFS and (2) determine the level of anthropic disturbance in the agroecosystem by comparing it to that of a nearby undisturbed rainforest fragment.

## Materials and methods

### Study site

This work was carried out at a rural property within the municipality of Araponga, Zona da Mata in Minas Gerais state, Brazil (20º 38´ 39.76´´ S, 42º 3´ 0.27´´W), located at an altitude of 1,315 m. The region’s climate is mesothermal and annual rainfall varies from 1,280 mm to 1,800 mm. The terrain is mountainous; declivity is between 20 and 45%, with prevalence of latosols. This rural property had three coffee plantations (*Coffea arabica* L. cv. Red Catuaí) using the agroforestry system and was close to an Atlantic rainforest fragment.

### History and characterization of the study areas

In the 1980s, this rural property was used for corn and bean cultivation and cattle raising. At that time, burning practices were frequent. The coffee plantations began in 1999. Chemical fertilizers were applied to the coffee tree plantations between 1999 and 2001. After that, chemical fertilizers were gradually replaced by organic fertilizers. In 2003, other species of trees were incorporated inside the coffee plantations, and since then, the transition from solely coffee plantations to AFS began. Farmers planted trees, especially fruit trees, randomly grown within the coffee plantations. Other management changes also occurred: farmers stopped weeding spontaneous growing plants, started tilling the soil, and stopped using pesticides. Currently, two coffee plantations are certified as organic by BCS Öko-Garantie, a German certification organization. One of the plantations is fertilized using poultry litter and the other one with cow manure. In a third plantation, a natural farming system (only plant residues are added to the tilled soil) was adopted and the crop has been certified by the Shumei Japanese organization of natural farmers.

The experimental design was carried out in coffee plantations using three different organic fertilizer treatments (natural fertilizer (NF), poultry litter (PL), cow manure (CM)) and in a fragment forest (FF). The 25,000 square meters of Atlantic forest fragment is considered as a remnant of the forest and does not present recent signs of anthropogenic disturbances, except for activities involving removal of fallen branches that were used for fertilizing natural coffee plantations.

The naturally fertilized coffee plantation was comprised of an area of 8,000 square meters, with 2.3 × 1.2 meters spacing between coffee trees. In this system, coffee trees were last pruned in 2011. Such plantations are mostly cultivated together with the following plant species: banana (*Musa* sp.); capoeira-branca (*Solanum argenteum*); royal palm (*Archontophoenix cunninghamiana*) and pinto peanut (*Arachis pintoi*). Plant residues already present in coffee plantations are used as fertilizers, including banana pseudostem, capoeira-branca trunks, pinto peanuts, and leaf litter from the nearby forest fragment. Fertilization is performed by placing the plant material in a circle on the soil around the coffee tree, and the coverage is based on the size of the crown. This is performed once in November and again 45 days later. In total, approximately 70 tonnes ha^-1^ of residues are used per year in the plantations.

The coffee plantation fertilized with poultry litter comprised an area of 4,000 square meters, with 1.5 × 3.0 m plant spacing. Fertilization was performed by placing the fertilizer on the soil in a circle around the coffee tree, and coverage was based on the size of the crown. This was carried out once in December and again after 45 days after the first application. A total of 44.5 tonnes ha^-1^ of composted poultry litter, obtained from neighboring farms, was used per year in this plantation. To compost poultry litter, this material was assembled in layers interspersed with manure and coffee bean peels from the intended plantation. The material was moistened at least once a week and turned in every 30 days. The entire process takes 90 days. The predominant tree species in the area are “guatambu” (*Aspidosperma polyneurum*); “capoeira-branca” (*Solanum argenteum*) and banana (*Musa* sp.). Except for the banana trees that are planted around the coffee tree plantations as a physical wind barrier, the other species were randomly distributed in the plantations.

The coffee tree plantation fertilized using composted cow manure comprised an area of 15,000 square meters, with 3.0 × 1.5 m plant spacing. Fertilization was performed by placing the fertilizer on the soil in a circle around the coffee tree, and coverage was based on the size of the crown. This was performed once in December and again after 45 days after the first application. In total, approximately 13.2 tonnes ha^-1^ of composted cow manure from the rural property was used per year in the plantation. For composting, the manure was heaped in a pile, moistened every week, and turned every 30 days. The entire process takes 90 days. In this area, the main tree species were avocado (*Persea gratissima*) and banana (*Musa* sp.).

### Soil analyses

Physical analyses of soil total porosity, microporosity, macroporosity, soil moisture, and chemical analyses of soil macronutrients, organic matter, C:N ratio, and pH from the four areas evaluated were carried out. In each experimental plot were samples were collected from the soil layer at a depth of 0–10 cm with the aid of a volumetric ring. The methods used in the physical and chemical soil analyses were according to Donagema et al. (2011). The collection of soil samples for physical and chemical analyses was simultaneous with the collection of samples for nematological analyses.

### Sampling, extraction, and identification of nematodes

**Figure 1:**
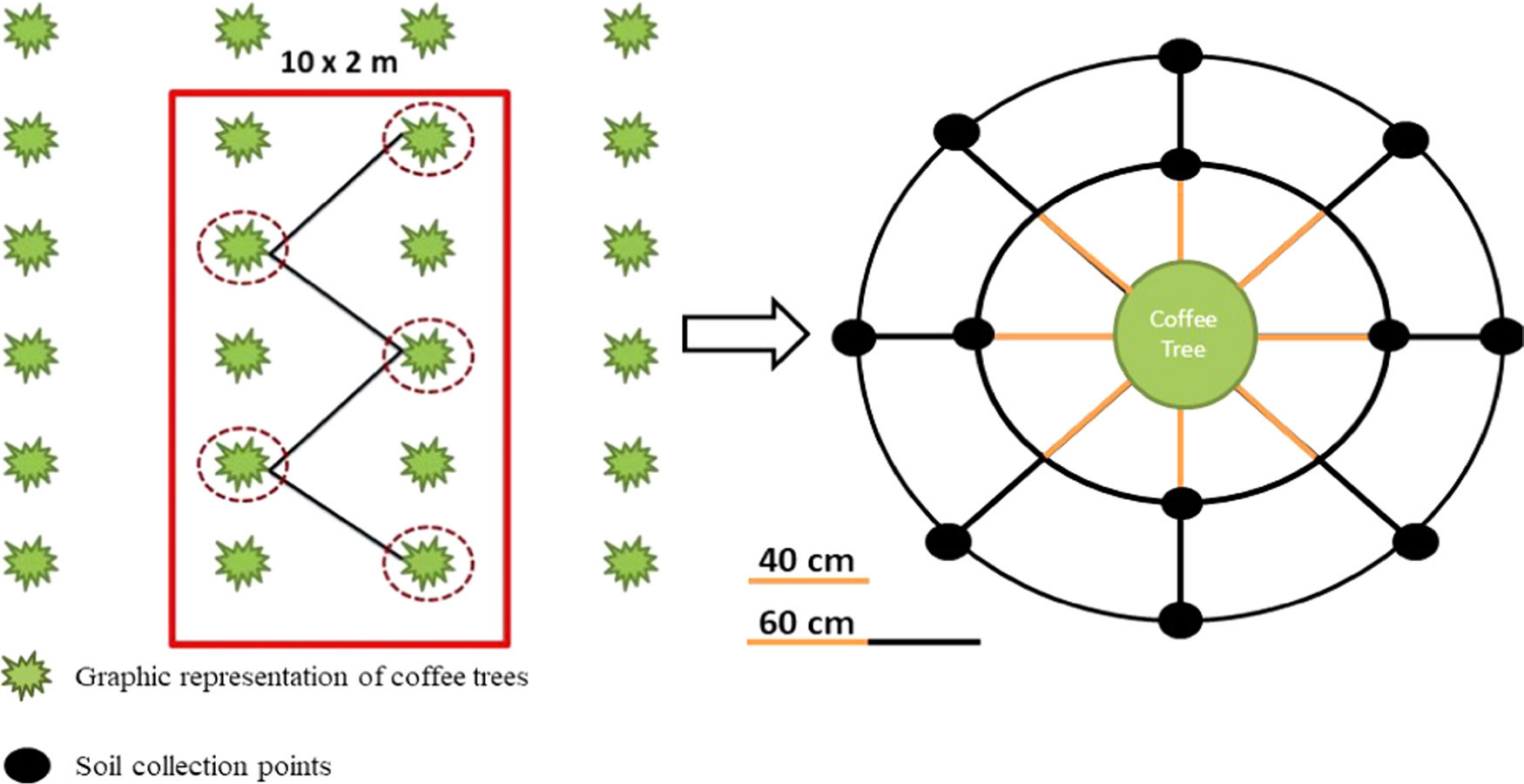
Sampling method for nematodes present in the soil in each coffee plantation.

Soil samples for estimation of nematode populations were collected from the three coffee plantations and the Atlantic forest fragment. For each fertilization treatment, five coffee trees were randomly selected and the soil at the base of the plant was sampled in a zigzag pattern. In each area, five soil samples were collected at a depth of 0–20 cm in September 2015 using a volumetric ring with a 2.5-cm diameter. A total of 12 samples were collected and homogenized as a mixed sample for each coffee tree selected (Huising et al., 2008) (Figure 1). Samples were then transferred into plastic bags, sealed, and labeled for subsequent extraction and identification of nematodes.

Extraction of nematodes was performed using the flotation method with a sucrose solution (Jenkins, 1964). Soil samples (100 cc) were first passed through a 2-mm mesh sieve to remove stones and roots. Sieved soils were then placed in a beaker containing 1,000 mL of water, and suspensions were manually stirred for a minute. Then, stirring was interrupted for 20 s so that thicker particles could settle and the supernatants were then transferred to a 400-mesh sieve. The retained material was then washed and transferred to Falcon tubes (50 mL) and centrifuged for 5 min at 1,008 × g, and the supernatants were discarded. Then, a sucrose solution (460 g of sucrose per liter of water) was added to the tubes, and the mixture was centrifuged for one minute at 112 × g. Suspensions were then passed through a 400-mesh sieve and washed under running water to remove the sucrose. Nematodes retained by the sieve were then transferred to Falcon tubes (50 mL) containing 10 mL of water. The tubes were kept in an incubator for 5 min at a temperature of 64°C, resulting in the death of the nematodes; however, the nematodes were not deformed by this process. Then, they were fixed by the addition of 1 mL of formaldehyde solution and stored at 4°C in a refrigerator.

Suspensions containing nematodes were pipetted into flat glass Petri dishes (60 diameter × 15-mm height) that were then placed under a binocular stereoscope (Bel Photonics®, model SZ) to count the nematodes. Nematodes were picked up using a needle and transferred to microscope slides (Precision Glass®, 26 × 76 mm) containing a drop of water + formaldehyde at a ratio of 10:1.

A double layer of matt varnish (Acrilex®) was applied to each slide to fix the coverslips in place (Paiva et al., 2006). To seal the space between the slide and coverslip, a double layer of colorless nail polish (Risqué®) was then applied. Nematodes were observed using an optical microscope (40 × objective) (Motic®-A210 model) and identified to the genus level using standard keys (Andrássy, 2005; Chaves et al., 1995; Jairajpuri and Wasim, 1992; Siddiqi, 2000), and classified into five trophic groups based on feeding habits: bacterivores, plant-parasites, fungivores, omnivores, and predators.

### Ecological parameters and indices of communities and ecosystems

The effect of fertilization on the nematode communities was assessed by calculating total abundance, trophic abundance, and relative abundance. Trophic abundance is the abundance of nematodes within the different trophic groups. Relative abundance was calculated as the percentage value of the number of genera that belong to a particular taxon or trophic group in relation to the total number of nematodes present in a sample using the following equation: *p*_*i*_ *=* *(n/N)* *×* 100, wherein *n* was the number of nematodes from each genus and *N* was the total number of nematodes in each sample (Cares and Huang, 2008).

To compare the diversity and dominance between the areas, the Shannon (H´) and Simpson (Ds) indexes were calculated. The Shannon index applies equal weight to rare and abundant genus (Shannon, 1948) using the equation *H´ = −∑ p*_*i*_ *× LN (p*_*i*_), wherein *p*_*i*_ is the relative abundance and *LN* is the logarithmic function. The Simpson index measures the probability of two randomly selected nematodes from a sample belonging to the same species. This index is calculated using the equation *Ds = ∑ (p*_*i*_*)²*, wherein *p*_*i*_ was the relative abundance of nematodes in a sample (Simpson, 1949).

The specific indexes for nematodes that were developed by Bongers (1990), Maturity (MI), Maturity 2–5 (MI 2–5), and Plant Parasites (PPI) indexes, and by Ferris (2010), Basal (BI), enrichment (EI), and structure (SI) indexes were calculated to obtain the environmental conditions of the areas studied.

To calculate the maturity indexes, all soil nematodes except plant-parasitic ones were considered. MI 2–5 was based on the calculations for the nematode groups that had a *c-p* value between 2 and 5; therefore, plant-parasitic and nematodes with a *c-p* value of 1 are excluded. The Maturity Index is considered a measure of environmental disturbance, and low MI values indicate disturbed and enriched environments while high MI values indicate stable environments (Bongers, 1990). Due to the functionality of plant-parasitic nematodes in the agroecosystem, this group is used to calculate the level of anthropic disturbance using PPI (Bongers, 1990). The equation used to calculate MI, MI 2–5 and PPI is *∑[v(i) × pi]*, where *v*(*i*) corresponds to the *c-p* value of the nematode family and *pi* the relative abundance of that family in the sample (Bongers, 1990).

The Basal Index (BI), Enrichment Index (EI), and Structure Index (SI) are based on the importance of functional guilds of nematodes as indicators and are descriptors of food web conditions. Basal food webs are those which are diminished due to stress, scarce availability of resources, contamination, or other harsh environmental conditions. Nematodes present in these food webs are represented by bacterial and fungal feeding taxa from the c-p2 class of the MI. Enriched food webs are those with high availability of resources due to the occurrence of a disturbance event. Opportunistic bacterial feeding nematodes from the c-p1 class are predominant in these food webs. Fungal feeding nematodes from the c-p2 class might increase when more complex resources, such as with a higher C:N ratio, becomes available, or when fungal feeding activity is enhanced in detriment to bacterial feeding activity. Structured food webs are those with more resources available. Nematode taxa from the c-p3 class are present in less structured food webs, while structure in the community will be greater when nematode taxa from the c-p4 and c-p5 classes are present. These indexes were obtained through the NINJA platform (Sieriebriennikov et al., 2014) and to characterize the conditions of the studied areas, the data were plotted according to Ferris (2010).

### Statistical analysis

The values obtained for the attributes from the physical and chemical soil analyses were submitted to analysis of variance (one-way ANOVA), and when the results were statistically significant, they were compared using the Tukey test (*p* < 0.05). The chemical attributes for phosphorus (P) and potassium (K) did not follow a normal distribution, therefore the averages obtained were submitted to non-parametric Kruskal–Wallis analysis, and compared using Dunn’s method (*p* < 0.05).

The calculated abundances, diversity, and dominance measures, and nematode indexes were submitted to analysis of variance, and when the results were statistically significant, they were compared using the Tukey test (*p* < 0.05). Genera-related abundance did not follow a normal distribution; therefore, the non-parametric Dunnett’s method was used in this case. Statistical analysis was performed using the SigmaPlot® 12.0 (Systat Software, Inc.) software.

## Results

The physical attributes of the soils did not differ among the areas (F_TP (3,19)_ *= *1.08, *P = *0.38; F_Micro (3,19)_ *= *1.55, *P = *0.24; F_Macro (3,19)_ *= *2.16, *P = *0.13; F_Micro (3,19)_ *= *2.93, *P = *0.06; Table 1). In relation to the chemical attributes, pH and nitrogen did not present any statistical differences (F_pH (3,19)_ *= *1.58, *P = *0.23; F_N (3,19)_ *= *1.35, *P = *0.29; Table 2). The macro-nutrient phosphorus (P) displayed higher values in PL and CN (H_(3,19)_ *= *17.65, *p* < 0.001), potassium was found at a higher concentration in PL (H_(3,19)_ *= *16.71, *p* < 0.001), whilst the level of organic material was higher in FF, PL and NF (F_(3,19)_ *= *20.74, *p* < 0.001). A higher ratio of C:N was observed in PL and CM (F_(3,19)_ *= *20.24, *p* < 0.001) (Table 2).

**Table 1.** Physical characterization of soil samples collected at a depth of 0–10 cm from the following areas in Araponga, Minas Gerais, Brazil: forest fragment (FF) and coffee plantations naturally fertilized (NF), fertilized with poultry litter (PL) or cow manure (CM).

	Physical attributes (%)				
	Textural class	TP	Micro	Macro	SM
NF	Clayey	56.02 ± 1.08	34.79 ± 0.99	21.23 ± 1.55	25.80 ± 1.43
PL	Clayey	59.23 ± 1.68	36.83 ± 0.90	22.40 ± 1.13	27.23 ± 0.94
CM	Clayey	58.51 ± 1.01	33.27 ± 1.22	25.26 ± 1.79	30.12 ± 1.18
FF	Clayey	59.34 ± 1.95	32.12 ± 2.75	27.21 ± 2.59	25.67 ± 1.08

Notes: * Macro, macroporosity; Micro, microporosity; SM, Soil moisture; TP, total porosity.

**Values are means ± s.e.m.; none of the results were significantly different using analysis of variance (*p* < 0.05).

**Table 2.** Chemical characterization soil sampled at a depth of 0–10 cm from the following areas in Araponga, Minas Gerais, Brazil: forest fragment (FF) and coffee plantations, naturally fertilized (NF), poultry litter fertilized (PL) and cow manure (CM) fertilized.

		N	P	K	OM	C:N
	pH	(%)	mg/dm^3^	mg/dm^3^	ppm	
NF	5.8 ± 0.05^ns^	0.27 ± 0.01^ns^	15.4 ± 0.42b	319 ± 1.21ab	17.65 ± 0.52a	25.65 ± 0.23b
PL	5.5 ± 0.08^ns^	0.28 ± 0.01^ns^	705.6 ± 6.74a	977 ± 0.79a	17.73 ± 0.63a	48.21 ± 9.60a
CM	5.6 ± 0.09^ns^	0.27 ± 0.01^ns^	46.7 ± 0.65a	329 ± 5.37ab	14.48 ± 0.35b	31.01 ± 6.20a
FF	5.7 ± 0.05^ns^	0.29 ± 0.01^ns^	3.0 ± 0.05b	60 ± 2.43b	19.46 ± 0.28a	39.01 ± 7.80ab

Notes: C:N = Carbon-nitrogen ratio, K = Potassium, N = total organic nitrogen, OM = Organic matter, P = available Phosphorus.

Values are means ± s.e.m.; ns: Non-significant by analysis of variance. pH, N, OM, and C:N ratio, Tukey Test; P and K Dunn’s method (*p *< 0.05).

A total of 2139 nematodes were collected and classified into the five trophic groups: plant-parasites, bacterivores, fungivores, omnivores, and predators (Table 3). No differences in total nematode abundance were found when comparing the evaluated areas (F_3,19_ *= *2.26, *P = *0.125).

**Table 3.** Average values (± standard error) for the total abundance of nematodes per 100 g of dry soil from an Atlantic forest fragment (FF), a naturally fertilized (NF) coffee plantation, poultry litter (PL) fertilized coffee plantation, and a coffee plantation fertilized with cow manure (CM).

Feeding habits	FF	NF	PL	CM
Bacterivores	108 ± 2.73b	170 ± 1.14b	380 ± 7.72a	378 ± 5.83a
Plant-parasites	270 ± 5.03a	287 ± 3.37a	183 ± 3.12b	128 ± 1.28b
Fungivores	84 ± 2.83a	51 ± 5.16a	32 ± 1.12b	13 ± 0.97b
Omnivores	11 ± 0.54^ns^	9 ± 0.37^ns^	2 ± 0.41^ns^	2 ± 0.83^ns^
Predators	10 ± 0.73^ns^	9 ± 0.37^ns^	8 ± 0.24^ns^	4 ± 0.25^ns^
Total	483 ± 7.20^ns^	526 ± 5.63^ns^	605 ± 9.09^ns^	525 ± 4.59^ns^

Notes: Values followed by the same letter on the same line did not differ when using the Tukey test (*p* < 0.05); ^ns^Non-significant by analysis of variance.

Regarding the trophic groups, the abundance of plant-parasitic nematodes was higher in the soil from naturally fertilized coffee plantations and the Atlantic forest fragment (F_3,19_ *= *18.58, *P = *0.0001) (Table 3). Bacterivorous nematodes were more abundant in plots that had been fertilized with composted poultry litter and cow manure, than plantations were natural fertilizer was used and in soil from the forest fragment (F_3,19_ *= *30.96, *P = *0.0001). Fungivorous nematodes were more abundant in soil from the forest fragment and the naturally fertilized plantation than in the other plantations (F_3,19_ *= *9.52, *P = *0.001). No differences were found with respect to omnivorous (F_3,19_ *= *4.40, *P = *0.019) and predatory nematodes (F_3,19_ *= *1.49, *P = *0.254) (Table 3).

We identified 23 nematode genera distributed into 21 families (Table 4). Bacterivorous nematodes from the genus *Acrobeles* were significantly more abundant in plantations fertilized with cow manure (H_3,19_ *= *9.15, *P = *0.02) as well as the genus *Rhabditis* which was also more abundant in areas where cow manure was used (H_3,19_ *= *17.86, *P = *<0.001). In the plant-parasitic trophic group, the genus *Criconema* (H_3,19_ *= *11.64, *P = *0.009) and *Pratylenchus* (H_3,19_ *= *12.86, *P = *0.005) were more abundant in the soil from the forest fragment and naturally fertilized coffee plantations. The genus *Helicotylenchus* was found in the highest abundance in naturally fertilized soil from coffee plantations (H_3,19_ *= *10.57, *P = *0.014). The highest relative abundance of the genus *Aphelenchus* was found in soil from the forest fragment (H_3,19_ *= *13.65, *P = *0.003).

**Table 4.** Mean relative abundance of nematode genera found in soil samples from an Atlantic forest fragment (FF), a naturally fertilized (NF) coffee plantation, a poultry litter (PL) fertilized coffee plantation, and cow manure (CM) fertilized coffee plantation.

Families	Genera	FF	NF	PL	CM
*Bacterivores*		*Areas*			
Alaimidae	*Alaimus*	0.01^ns^	0.02^ns^	0.00^ns^	0.00^ns^
Bunonematidae	*Bunonema*	0.00^ns^	0.00^ns^	0.01^ns^	0.00^ns^
Cephalobidae	*Acrobeles*	0.09b	0.10b	0.10b	0.18a
	*Cephalobus*	0.05^ns^	0.04^ns^	0.02^ns^	0.02^ns^
Diplogastridae	*Diplogaster*	0.00^ns^	0.00^ns^	0.03^ns^	0.01^ns^
Panagrolaimidae	*Panagrolaimus*	0.00^ns^	0.01^ns^	0.03^ns^	0.01^ns^
Plectidae	*Plectus*	0.01^ns^	0.01^ns^	0.02^ns^	0.02^ns^
	*Wilsonema*	0.00^ns^	0.01^ns^	0.00^ns^	0.01^ns^
Prismatolaimidae	*Prismatolaimus*	0.01^ns^	0.01^ns^	0.00^ns^	0.00^ns^
Rhabditidae	*Rhabditis*	0.05b	0.12b	0.38a	0.49a
Teratocephalidae	*Teratocephalus*	0.00^ns^	0.01^ns^	0.02^ns^	0.00^ns^
*Plant-parasites*		*Areas*			
Anguininae	Ditylenchus*	0.03^ns^	0.02^ns^	0.04^ns^	0.01^ns^
Criconematidae	*Criconema*	0.15a	0.08a	0.02b	0.01b
Hoplolaimidae	*Helicotylenchus*	0.21b	0.35a	0.20b	0.17b
Longidoridae	*Longidorus*	0.03^ns^	0.01^ns^	0.02^ns^	0.02^ns^
Pratylenchidae	*Pratylenchus*	0.07a	0.04a	0.01b	0.00b
Trichodoridae	*Trichodorus*	0.02^ns^	0.02^ns^	0.00^ns^	0.00^ns^
Tylenchidae	*Tylenchus*	0.05^ns^	0.03^ns^	0.04^ns^	0.02^ns^
*Fungivores*		*Areas*			
Aphelenchidae	*Aphelenchus*	0.14a	0.07b	0.05b	0.02b
Diphtherophoridae	*Diphtherophora*	0.02^ns^	0.01^ns^	0.00^ns^	0.00^ns^
Tylencholaimellidae	*Tylencholaimellus*	0.01^ns^	0.01^ns^	0.00^ns^	0.00^ns^
*Onivores*		*Areas*			
Dorylaimidae	*Dorylaimus*	0.03^ns^	0.02^ns^	0.00^ns^	0.00^ns^
*Predator*		*Areas*			
Mononchidae	*Mononchus*	0.02^ns^	0.02^ns^	0.01^ns^	0.01^ns^

Notes: Values followed by the same letter on the same line did not differ when using Dunnett’s method (*p* < 0.05).

*Ditylenchus specimens found in soil can be plant-parasitic and/or fungivorous.

Regarding diversity and dominance of the studied areas, significant differences were detected both in the Shannon (F_3,19_ *= *12.17, *P = *<0.001) and Simpson (F_3,19_ *= *82.36, *P = *<0.001) indexes. In the plantation fertilized with the cow manure, the diversity was lower (1.95) and dominance was greater (0.33) than other areas studied (Table 5).

**Table 5.** Average values (± standard error) of Shannon and Simpson indices of nematode communities in soil samples from an Atlantic forest fragment (FF), a naturally fertilized (NF) coffee plantation, a poultry litter (PL) fertilized coffee plantation, and cow manure (CM) fertilized coffee plantation.

	Areas evaluated			
Diversity indexes	FF	NF	PL	CM
Shannon (H’)	2.34 ± 0.03a	2.24 ± 0.03a	2.09 ± 0.04a	1.95 ± 0.06b
Simpson (Ds)	0.14 ± 0.002b	0.17 ± 0.006b	0.20 ± 0.002b	0.33 ± 0.002a

Notes: Values followed by the same letter on the same line did not differ when using the Tukey test (*p* < 0.05).

**Figure 2:**
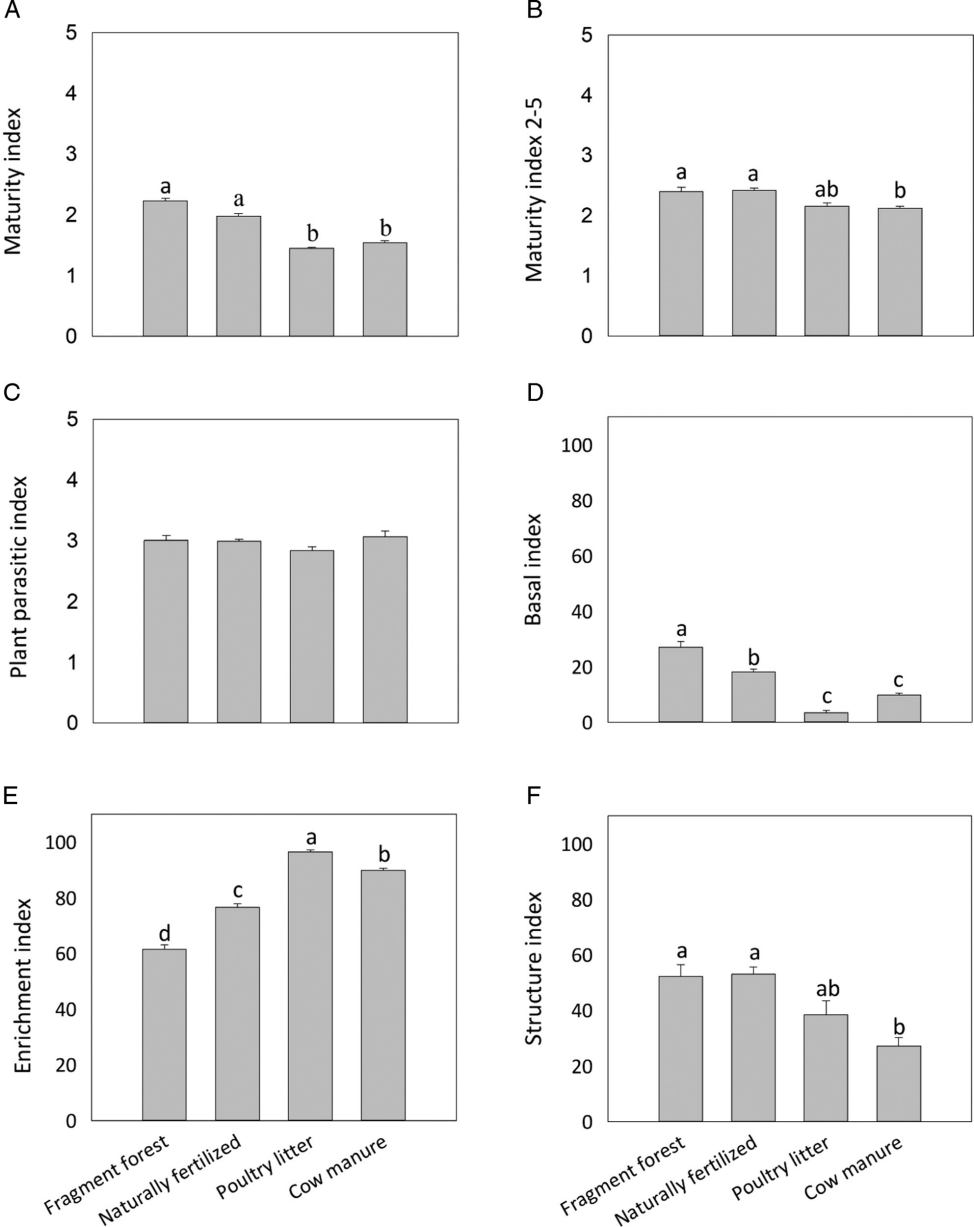
Average values (± standard error) of (a) the maturity, (b) the maturity 2–5, (c) the plant parasitic, (d) the basal, (e) the enrichment, and (f) the structure indexes. Values followed by the same letter did not differ when using the Tukey test (*p* < 0.05).

The results for the maturity index (MI) showed significant differences (F_3,19_ *= *122.05, *P = *<0.001, Figure 2A), with higher values in the forest fragment (2.24) and in the naturally fertilized plantation (1.98), whilst lower values were seen in the soils from plantations where cow manure (1.54) and poultry litter (1.45) had been employed. The maturity index 2–5 (MI 2–5) also a showed significant difference (F_3,19_ *= *7.75; *P = *0.002, Figure 2B), where the poultry litter fertilized plantation showed a lower value (2.16) than the other areas. Regarding the plant-parasitic nematode index (F_3,19_ *= *1.99; *P = *0.157), no differences were observed between the areas (Figure 2C).

Significant differences were detected in the basal index (F_3,19_ *= *79.75, *P = *<0.001, Figure 2D), enrichment index (F_3,19_ *= *172.65, *P = *<0.001, Figure 2E), and structure index (F_3,19_ *= *122.05, *P = *<0.001, Figure 2F). The basal index was highest in the fragment forest, followed by the naturally fertilized plantation and the lowest values were seen in the areas fertilized with cow manure and poultry litter (Figure 2D). The enrichment index showed higher values in soil from the coffee plantation fertilized with poultry litter, followed by cow manure, naturally fertilized plantation, and the fragment forest (Figure 2E). The forest fragment, natural coffee plantation, and poultry litter plantation had the highest structure index. Coffee plantations fertilized with cow manure had the lowest value, but did not differ from plantations fertilized with poultry litter (Figure 2F).

**Figure 3:**
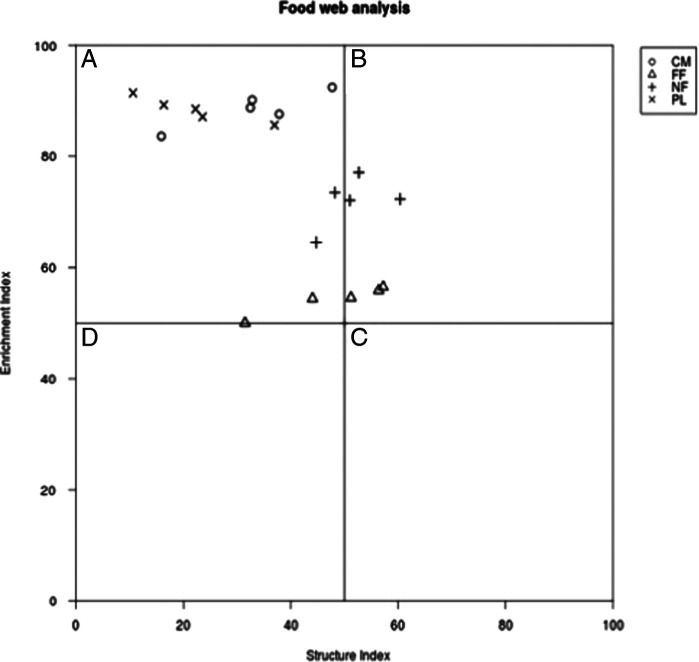
Food web analysis of coffee crops fertilized with cow manure (CM), poultry litter (PL), plant residues (NF), and in a forest fragment (FF). Quadrant A: enriched and structured agroecosystem; quadrant B: mature and structured environment; quadrant C: mature and stable environment, fertile soil; and quadrant D: degraded and depleted soil. (Ferris, 2010).

The faunal profile data, observed in quadrant A, indicated that the plantations fertilized with poultry litter and cow manure presented a soil enrichment profile and this was an indication of an agroecosystems with a high level of ecological disturbance. The naturally fertilized coffee plantation, observed in quadrant B, presents similar food web characteristics to the forest fragment (Figure 3).

## Discussion

The total abundance of nematodes when comparing the coffee AFS and the Atlantic rainforest fragment indicated that there were similarities in the agroecosystem communities. This result was also observed in similar studies (Franco-Navarro and Godinez-Vidal, 2017; McQueen and Treonis, 2020). Even if soil fertility practices are used, these disturbances are not enough to change the distribution of nematode communities in agroecosystems (Marinho et al., 2014). The presence of the trees in the coffee plantations is an important factor that contributes to regulating soil temperature, reducing exposure of coffee plants to direct sunlight, and, consequently, making the environment more stable and similar to natural ecosystems (Gomes et al., 2016).

Plant-parasitic and bacterivorous nematodes are usually the dominant trophic groups in natural ecosystems and agricultural plantations (Grabau et al., 2019; Hu et al., 2014). They often exhibit a negative relationship in the soil (Liu et al., 2016), because of the release of compounds and organic acids combined with high nitrogen levels present in fertilizers of animal origin, which inhibit the abundance of plant-parasitic nematodes (Oka, 2010). On the other hand, bacterivores are favored by the incorporation of animal manure in the soil, leading to increased food resources for bacterial feeders, which contribute to the decomposition of organic matter (Jiang et al., 2013).

The abundance of fungivores is favored by organic matter with high concentrations of lignin and cellulose (organic carbon sources) (Liu et al., 2016) (Table 3). These compounds are commonly found in plant residues from the forest litter, including branch and trunk residues. The forest litter used in the soils of naturally fertilized coffee tree plantations contains minor levels of lignin and cellulose because it has been partially decomposed or is mostly comprised of leaf residues. Fungivorous nematodes are sensitive to ammoniacal acids released in the soil by compounds with a low C:N ratio (Oka, 2010), justifying the lower abundance of this trophic group in the soil fertilized by poultry litter and cow manure.

The genus *Rhabditis* was present in the greatest abundance in soils fertilized using poultry litter and cow manure, and the higher abundance of *Acrobeles* was observed in coffee plantation fertilized with cow manure. These genera belong to groups of opportunistic nematodes and rapidly respond to the incorporation of organic fertilizers from animal origin in the soil and are usually more abundant in agricultural areas (Brmež et al., 2018; Carron et al., 2015; Van den Hoogen et al., 2020). The abundance of members of this family contribute to the mineralization and regulation of nutrients such as nitrogen, which is then available for the trees (Ferris et al., 1998; Sãnchez-Moreno et al., 2011).

The genera *Criconema* is a plant-parasitic nematode that is very sensitive to environmental disturbances, and its population is favored by soils covered with plants and in those with a high content of organic matter, such as natural vegetation and agroecosystems with low anthropogenic disturbance (Caixeta et al., 2016). The genus *Helicotylenchus*, which is abundant in the soil of naturally fertilized coffee plantations and the forest fragment, is usually a very common plant-parasitic nematode and is present at high densities in annual and perennial plantations and natural ecosystems (Tomazini et al., 2008). As expected, considering the source of the residues used to fertilize the soil of the coffee plantations, the results indicated a greater similarity between soils of naturally fertilized plantations and the Atlantic forest fragment. The presence of these plant-parasites, as well as other opportunistic trophic groups, is favored by a high organic matter content and high root density (Ritzinger et al., 2010). The genus *Aphelenchus* belongs to the fungivores trophic group and is associated with soils containing high recalcitrant organic matter levels, abundant in habitats with advanced stages of ecological succession (Niles and Freckman, 1998; Porazinska et al., 1999).

The crop fertilized with cow manure showed less diversity and greater genera dominance, for example like *Rhabditis*. This area had the lowest density of trees among the agroecosystems studied. These results may be associated with the greater availability of litter in the soil, which favors the abundance, diversity, and richness of communities of soil organisms (Moço et al., 2009).

MI values close to two, as found in the forest fragment (2.23) soil and the soil from the naturally fertilized coffee (1.98) plantation (Figure 2), indicated that these environments were in an ecological succession stage. Values between one and two, as obtained for the soils of coffee crops fertilized with animal manure (CM * = * 1.43, PL* = *1.41), indicated that these agroecosystems present high ecological disturbance due to the enrichment of the soil with organic fertilizers (Bongers, 1990; Bongers and Ferris, 1999). The lowest values for MI 2–5 and the basal index were found in crops fertilized with poultry litter, suggesting a food web dominated by bacteria under nutrient enrichment conditions (DuPont et al., 2009). The similarity of the PPI between areas may be associated with the high C/N ratio found in agroecosystems soils. The lower N availability causes plants to produce less root volume, thus reducing the availability of resources for plant-parasitic nematodes (Ugarte et al., 2013).

The SI combined with EI indicated that coffee crops fertilized with poultry litter and cow manure have similar characteristics. According to Ferris (2010), these agroecosystems have soils with moderate disturbance characteristics, enriched with nitrogen, and with a balanced decomposition channel, although with bacterial decomposition. The soil of the forest fragment and the naturally fertilized coffee plantation showed low disturbance, with a food web characteristic of mature soil. The position near quadrant C indicated that the ecosystem is close to an undisturbed environment, with a decomposition channel dominated by fungi (Figure 2). These results also suggest that the soil condition of the crops is associated with the way they are managed. Agroecological management without the use of chemical inputs (fertilizers and pesticides), no soil disturbance, constant employment of organic residues from trees, and management of spontaneous herbaceous vegetation, favored the quality of the soils of agroecosystems (Mulder et al., 2003).

According to the faunal profile results, it can be inferred that the application of animal waste fertilizers stimulated the bacterial channel. Although they are characterized as disturbed agroecosystems, it can be stated that due to the biodiversity present in AFS, these areas in the long term may be closer to quadrant B, which indicates an environment with lower ecological disturbance (Ferris, 2001). Furthermore, the faunal profile confirms the hypothesis that there is a similarity between the naturally fertilized coffee plantation and the Atlantic rainforest fragment.

The nematodes communities present in the soil of the studied areas showed the similarity between the agroforestry systems and the natural ecosystem (forest). The systems presented low levels of ecological disturbance when compared to forest fragments.
